# Associations of Mentally Active Versus Passive Sedentary Behavior with Overweight/Obesity in Adults: Role of Patterns and Sex

**DOI:** 10.3390/healthcare13040361

**Published:** 2025-02-08

**Authors:** Daliya S. Alobaid, Abdullah B. Alansare

**Affiliations:** Department of Exercise Physiology, College of Sport Sciences and Physical Activity, King Saud University, King Khalid Rd, Riyadh 80200, Saudi Arabia; aalansare@ksu.edu.sa

**Keywords:** body mass index, sitting, cognitive function, women, men, isotemporal substitution

## Abstract

**Objectives**: This study assessed associations of total and mental-activity-based sedentary behavior (SB) with the risk of being overweight or obese in adults. The role of sex and SB patterns and the effects of statistically exchanging different mental-activity-based SBs on body mass index (BMI) were explored. **Methods**: Participants (n = 1154) from the Saudi Post-COVID-19 Sedentary Behavior Survey self-reported demographics, health-related variables, and last-measured body height and weight. BMI was calculated to classify adults as normal weight or overweight/obese. The Sedentary Behavior Questionnaire estimated total and mentally active and passive SB per day, during weekdays, and on weekends. Adjusted logistic regression and isotemporal substitution models assessed the associations of SBs with the risk of being overweight or obese and the effects of displacing mental-activity-based SBs on BMI, respectively. **Results**: No significant relationships were observed in the overall sample (*p* > 0.05). Once sex was considered, the risk of being overweight or obese increased between 5.2% and 10.7% for each hour increase in total and mentally active SBs per day and on weekends in men only (*p* < 0.05 for all). Replacing one hour of mentally active SB with mentally passive SB resulted in non-significant effects on BMI (*p* > 0.05). **Conclusions**: These findings reveal the importance of distinguishing between SBs based on mental activity for more optimal obesity outcomes, particularly in men. Importantly, exchanging mental-activity-based SBs does not appear to be an effective behavioral strategy to reduce obesity. Obesity prevention and management plans should target reducing total and mentally active SBs daily and on weekends.

## 1. Introduction

Obesity, which is an excessive and abnormal accumulation of body fat, has become a global pandemic [1]. The World Obesity Federation predicted that by 2030, one billion people will be living with obesity [2]. This prevalence is particularly anticipated to be higher in females than males (i.e., one in five women vs. one in seven men) [2]. Moreover, this alarming estimation is projected to increase the global economic burden such that the financial cost of obesity will exceed $3 trillion by 2030 [3]. In addition to being a significant risk factor for many non-communicable diseases and mortality [4], obesity is now recognized as an independent chronic disease [5]. This disease is a complex, multifactorial condition and can be triggered by several lifestyle factors, such as physical inactivity, an unhealthy diet, and sedentary behavior (SB) [3,6]. As a result, lifestyle modifications have been proposed to confront obesity and its ramifications [6].

Of particular interest, SB is defined as any activity that occurs while awake and costs ≤1.5 metabolic equivalent of energy expenditure [7]. A recent synthesis of global large-scale and population-based investigations evaluated the prevalence of SB and found that adults spend a median of 8.2 h/day in SB [8]; men tended to have higher levels of SB compared to their counterbalanced women [9]. Moreover, consistent evidence showed that a higher level of SB was associated with an increased risk of cardiovascular disease, diabetes, mental disorders, and obesity [10,11], independent of physical activity and diets [12,13]. On top of the vital health hazards that SB induces, recent reports revealed a significant financial burden of SB on the Canadian and Finnish economies (i.e., $2.2 and $1.5 billion, respectively) [14,15]. Hence, SB reduction interventions may be a crucial lifestyle strategy to manage the accelerated growth in the prevalence of obesity and SB and its impacts on public health and economies.

It is worth noting that preliminary studies indicate distinctive associations of various types and patterns of SBs with health outcomes [11,16]. For instance, increased mentally passive SB (i.e., any SB that does not require higher cognitive function such as TV viewing) was associated with a greater risk of psychological distress [17], poor mental performance [18], and overweight or obesity [19]; however, no comparable associations were observed with higher levels of mentally active SB (i.e., any SB that requires higher cognitive function, such as reading time) [17,19]. Furthermore, increased overall SB on weekends, but not during weekdays, was associated with an increased likelihood of anxiety, depression, and obesity in adults [20,21]. Whether mentally active versus passive SB during weekdays and on weekends has different associations with obesity in men and women remains overlooked. Assessing these relationships is necessary for achieving more comprehensive public health policies to reduce obesity and SB and optimize public health. These assessments become even more pivotal as the current global SB recommendations for adults suggest reducing SB for favorable overall and metabolic health, without distinguishing between its types and patterns [22,23].

Therefore, the main aim of this investigation was to examine the associations of total, mentally active, and mentally passive SB with the risk of being overweight or obese and to assess the role of sex and SB patterns. An exploratory aim was to evaluate whether statistically exchanging time spent in mentally active and passive SB would lead to a more favorable obesity outcome. It was hypothesized that total and mentally passive SB would be associated with a greater risk of being overweight or obese than mentally active SB, particularly on weekends.

## 2. Materials and Methods

This secondary analysis used data from the Saudi Post-COVID-19 Sedentary Behavior Survey (SPSB) [16,24]. The study was executed following the principles of the Declaration of Helsinki. The study’s protocol was reviewed and approved by the Institutional Review Board at King Saud University (No: KSU-HE-22-773). The manuscript was reported by adhering to the Strengthening the Reporting of Observational Studies in Epidemiology (STROBE) guidelines.

### 2.1. Recruitment and Data Collection

**Figure 1:**
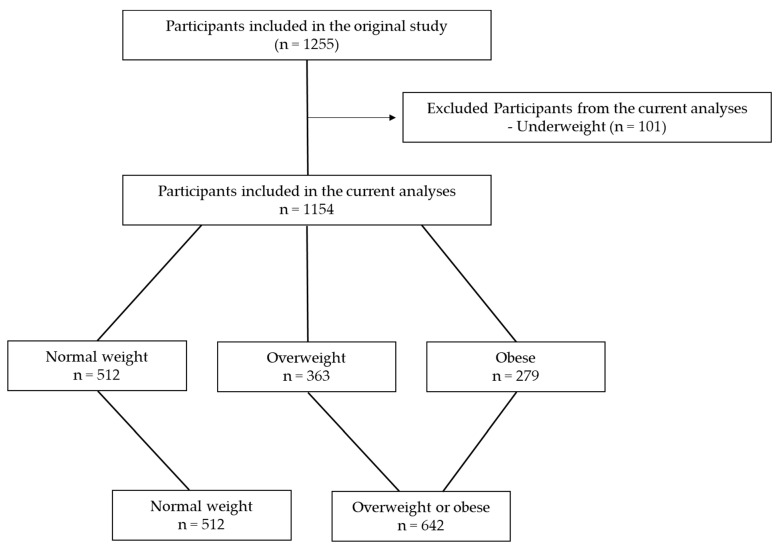
Flowchart of participants who were included in the current study.

The recruitment and data collection were described in detail in the original study [24]. Briefly, the SPSB was a web-based, cross-sectional investigation that estimated the prevalence of various SBs post-COVID-19 in Saudi adults. The demographics and health-related variables, anthropometric values, and SBs were self-reported. Data collection started on 2 December 2022 and was concluded on 25 January 2023. The sampling method was convenience sampling, where participants (n = 1255) were invited through social media platforms (e.g., WhatsApp and X) and King Saud University’s official emails. Considering that the current analysis focuses on the risk of obesity, only adults with normal weight (n = 512), overweight (n = 363), or obesity (n = 279) were included, and those with underweight were excluded (n = 101) (Figure 1). Thus, the total sample included in this investigation was 1154 participants.

### 2.2. Measurements

#### 2.2.1. Demographics and Health-Related Variables

Demographics and health-related variables including age, sex, education, occupation, smoking status, and having a chronic disease were self-reported using standardized questions (Table 1). The participants (n = 1154) tended to be healthy, non-smokers, young-to-middle-aged adults, and currently working or studying. In addition, slightly over half of the participants were female adults (53.6%), with overweight or obesity (55.6%), and spent a median of 8.4 h/day in total SB. Men appeared to accumulate more mentally active SB than women (6.3 vs. 5.2 h/day, respectively).

#### 2.2.2. Anthropometric Values

Each participant was asked to report their most recent body weight (in kilograms [kg]) and height (in centimeters). Body mass index (BMI) was then calculated using the following standardized equation: BMI = body weight (in kg)/body height (in meters squared [m^2^]) [25]. Then, the World Health Organization BMI classification was utilized to categorize the participants into different obesity levels [25]. The participants were classified as underweight (BMI < 18.5 kg/m^2^), normal weight (BMI = 18.5 to 24.9 kg/m^2^), overweight (BMI = 25 to 29.9 kg/m^2^), or obese (BMI ≥ 30 kg/m^2^). Given that this investigation addresses the risk of obesity, participants with overweight or obesity were grouped into one category and participants with normal weight were assigned to another category.

### 2.3. Sedentary Behavior

SB was estimated using the Sedentary Behavior Questionnaire [SBQ] [26,27]. The validity of the SBQ in adults was confirmed by comparing it with accelerometers and the International Physical Activity Questionnaire [26,27]. The reliability of the SBQ in adults was also previously established using test–retest methods [26,27]. The SBQ estimates time spent in 9 mentally active or passive SBs during a weekday and, separately, on a weekend day. Mentally active SB included the following items: playing computer or video games, playing a musical instrument, sitting and reading a book or magazine, doing artwork or crafts, sitting and talking on the phone, doing paperwork or computer work, and sitting and driving a car, bus, or train [11]. The duration of these mentally active SBs during a weekday were aggregated to estimate mentally active SBs during a weekday (hours/day). Likewise, the sum of these mentally active SBs on a weekend day was used to calculate mentally active SBs on a weekend day (hours/day). Afterward, overall mentally active SB per day (hours/day) was computed as follows: [(mentally active SB during a weekday × 5) + (mentally active SB on a weekend day × 2)]/7 [26].

On the other hand, the SBQ contained the following mentally passive SBs: sitting and listening to music and watching TV [11]. The total time spent in these mentally passive SBs during a weekday was summed to estimate mentally passive SBs during a weekday (hours/day). Comparably, the sum of these mentally passive SBs on a weekend day was utilized to calculate mentally passive SBs on a weekend (hours/day). Subsequently, overall mentally passive SB per day (hours/day) was computed as follows: [(mentally passive SB during a weekday × 5) + (mentally passive SB on a weekend day × 2)]/7 [26].

Lastly, the total SB during a weekday (hours/day) was estimated by summing times spent in all SBs during a weekday. The total SB on a weekend day (hours/day) was also estimated by aggregating the durations of all SBs on a weekend day. Ultimately, the overall total SB per day (hours/day) was computed as follows: [(total SB during a weekday × 5) + (total SB on a weekend day × 2)]/7 [26].

### 2.4. Statistical Analysis

Demographic, anthropometric, and health-related measures of the participants were reported as means and standard deviations (SD), medians and interquartile range (IQR), or frequency (n) and percentage (%). Three types of regression models were fitted to address the study’s hypotheses as follows. First, multiple linear regression models were fitted with BMI (a continuous variable) as the outcome, SBs as predictors, and demographic and health-related measures as covariates. This analysis was performed to determine significant confounders that should be adjusted for. Then, binary logistic regression models adjusted for confounders (i.e., age, sex, occupation, and chronic disease status) evaluated whether higher total, mentally active, or mentally passive SB would be associated with a higher risk of obesity in the overall sample and, separately, for each sex. Lastly, isotemporal substitution regression models adjusted for confounders were fitted to assess whether replacing one hour of mentally active and passive SB would be associated with a lower risk of obesity for the whole sample and, separately, for each sex. The statistical analyses were performed using JASP software (JASP 0.15 Version, Amsterdam, The Netherlands). A *p*-value <0.05 was considered significant.

## 3. Results

Table 2 presents the associations of total, mentally active, and mentally passive SB with the risk of overweight or obesity in adults. No significant relationships were observed between SBs and the likelihood of being overweight or obese in adults (*p* > 0.05 for all). Once sex was considered, significant associations were detected in men only; the risk of being overweight or obese increased by 5.2% and 7.6% for each hour increase in total SB per day and on weekends (*p* < 0.05 for both), respectively. Moreover, the likelihood of being overweight or obese increased by 7.5% and 10.7% for each hour increase in mentally active SB per day or on weekends (*p* < 0.05 for both), respectively. No other relationships were found.

Table 3 displays the isotemporal associations of mentally active and passive SBs with BMI in adults. Statistically replacing one hour of mentally active SB with mentally passive SB per day during weekdays or on weekends resulted in non-significant changes in BMI in the overall sample, women, or men (*p* > 0.05 for all).

## 4. Discussion

This investigation uniquely examined the associations of total, mentally active, and mentally passive SB with the risk of being overweight or obese in adults and evaluated the role of sex and SB patterns. Moreover, the effects of statistically replacing one hour of mentally active and passive SB on BMI were explored. It was found that only higher total and mentally active SBs per day and on weekends were associated with a higher likelihood of being overweight or obese, specifically in men. Moreover, statistically replacing one hour of mentally active SB with mentally passive SB did not improve BMI. These findings reveal the importance of distinguishing between SBs based on mental activity for more optimal adult obesity outcomes. Endeavors to confront obesity through SB reduction should target both total and mentally active SBs per day and on weekends, particularly in men. Importantly, exchanging mentally active and passive SB does not appear to be an effective behavioral strategy to reduce the risk of being overweight or obese in adults.

Preliminary research findings suggested that different SBs have unique relationships with health outcomes [17,18,19]. In line with this, the current study detected a greater likelihood of being overweight or obese in men only when total or mentally active SB was accumulated. Contrary to this observation, a previous investigation found that only increased mentally passive SB was related to a higher risk of being overweight or obese in older adults [19]. These discrepancies may theoretically be explained by complex interactions between age, psychological, and cultural differences. To illustrate, most participants in the current study were young-to-middle-aged adults and accumulated higher levels of mentally active than mentally passive SB [16]. This demographic usually experiences higher psychological stress stimulated by mentally active SB, such as studying or working on a computer [28,29]. They also spend more time in technology-based mentally active SBs [30] and are more likely to consume unhealthy snacks while engaging in these mentally active SBs [31,32]. Conversely, the participants in the previous study were older adults who accumulated more mentally passive than mentally active SB [19]. This age group commonly spends more time in solitary mentally passive SB (e.g., TV viewing), accompanied by unhealthy snack consumption [33,34] and a reduction in metabolic rate as they age [35]. Together, these factors contribute to longer times of mentally active SB for young-to-middle-aged adults and more mentally passive SB for older adults, increasing the risk of obesity and leading to distinct associations with obesity outcomes. Yet, this complex hypothesis warrants further examination in future research.

This study also provided insightful viewpoints, showing that the associations of total and mentally active SB with the risk of being overweight or obese were generally similar between men and women. However, these associations were only significant for men but not for women. This finding aligns with the existing literature, which suggests that SB is more strongly linked to obesity outcomes in men compared to women. For instance, prior investigations indicated that mentally active SB (i.e., video gaming or occupational SB) was associated with an increased risk of having a higher BMI, waist circumference, and waist-to-hip ratio in men [36,37]. At the same time, these associations were not detected in women [36,37]. These differences observed between men and women could be attributed to variations in SB patterns and lifestyle choices. Specifically, men tended to spend significantly more time in total or mentally active SB compared to women [16,37]. In addition, men are more likely to adopt poor diets and smoke while also accumulating higher SB than women [38]. The combination of longer times of SB alongside these unhealthy lifestyle choices may increase the risk of being overweight or obese in men but not in women. Still, further comprehensive investigations are needed to validate this hypothesis.

The current study also underlined the significant role of SB patterns in the links between SB and obesity outcomes in adults. In particular, it was found that total and mentally active SB on weekends, but not during weekdays, is related to a higher risk of being overweight or obese in men. This aligns with existing evidence suggesting that the correlations between SB and obesity outcomes are more pronounced on weekends than during weekdays [20]. Variations in sitting durations and social factors between weekdays and weekends may explain these pattern-related disparities. For example, adults often engage in more social SB on weekends, leading to a greater accumulation of total and prolonged bouts of SB on weekends compared to weekdays [39,40]. These findings suggest that obesity prevention and management plans should not only focus on reducing daily SB but also targeting SB on weekends.

A novel aspect of this study was evaluating the effects of statistically exchanging one hour of mentally active and mentally passive SB on BMI in adults using isotemporal substitution models. The results showed no significant effects on BMI in the overall sample and, separately, in men or women. Only one prior study assessed the impacts of displacing different SBs on BMI in children [41]. It was found that replacing 30 min of SBs (i.e., reading, excluding that for academic-related purposes, sitting and talking, and listening to music) with an equivalent amount of leisure screen time or academic-related SBs led to heightened BMI in children [41]. On the contrary, a robust systematic scoping review demonstrated that replacing SB with an equivalent amount of light-, moderate-, or vigorous-intensity physical activity yielded substantial improvements in BMI, body fat percentage, and waist circumference across all ages [42]. These findings suggest that efforts to reduce SB should focus on replacing SB not with other SBs but with different physical activity intensities to promote favorable outcomes related to obesity in adults.

### 4.1. Strengths and Limitations

The current investigation has strengths that are worth mentioning. The data collection was performed using a web-based survey. This technique allowed the widespread reach of Saudi adults from different regions, resulting in a large sample size recruitment. This aspect would ultimately improve the generalizability of the current findings [43]. In contrast to most existing studies [44], the utilized SB assessment tool (i.e., SBQ) in the current investigation simultaneously estimated a wide spectrum of mentally active and passive SB and the patterns of SB (i.e., weekdays vs. weekends). As such, the SB measurement was comprehensive, permitting a thorough examination of the relationships between SBs and obesity outcomes.

Still, a few limitations exist and should be considered when interpreting the current results. First, sampling biases (e.g., certain participant groups self-select to be enrolled or not) and lower data quality (e.g., participants are prone to interruptions and distractions while completing the survey) are common challenges in web-based studies [43]. In addition, SB and body height and weight were self-reported, which could have introduced recall bias into the data [45]. Another important limitation is not measuring and adjusting for moderate-intensity physical activity (MVPA), an established confounder between SB and BMI [46]. These potential constraints could have influenced the associations observed between SB and obesity outcomes.

### 4.2. Clinical Significance

The current study sheds light on an overlooked aspect of obesity prevention and management plans. The findings suggest differentiating between SBs based on mental activity and focusing on plummeting total and mentally active SB, especially on weekends, for more effective obesity prevention in men. Hence, health professionals are encouraged to implement SB reduction in obesity prevention and management strategies for adults. Importantly, this reduction in total and mentally active SB should not be achieved by replacing it with other types of SB. In line with these findings, the existing physical activity and SB guidelines for adults recommend increasing physical activity and reducing overall SB for holistic health, including better obesity outcomes [23,47]. To complement these guidelines, the current study provides further evidence to reframe SB recommendations to prevent and manage obesity by specifically targeting the reduction in total and mentally active SB, particularly in men. Yet, further rigorous prospective research and randomized controlled trials are warranted to confirm the current findings.

## 5. Conclusions

In short, this study mainly examined the associations of SB with the likelihood of being overweight or obese while differentiating between various SBs based on mental activity and accounting for the role of sex and SB patterns. Although no significant associations were observed for the overall sample or women, higher mentally active SB was significantly associated with a higher risk of being overweight or obese in men. The relationships observed were more apparent on weekends. These findings draw attention to the significance of targeting total and mentally active SB and account for SB patterns when planning obesity prevention and management programs for adults. Importantly, while replacing mentally active SBs with other SBSs may not be an effective behavioral strategy for reducing the risk of being overweight or obese, displacement with different physical activity intensities may be a promising approach.

## Figures and Tables

**Table 1:** Demographic, anthropometric, and health-related measures of the participants (n = 1154).

Characteristic	Mean ± SD/Median (IQR)/n (%)
Age	
20 to 29 years old	546 (47.3%)
30 to 39 years old	307 (26.6%)
40 to 49 years old	193 (16.7%)
50 years old or older	108 (9.4%)
BMI (kg/m^2^)	27.0±6.4
BMI Classifications	
Normal weight	512 (44.4%)
Overweight or obese	642 (55.6%)
Sex	
Male	535 (46.4%)
Female	619 (53.6%)
Education	
Undergraduate or less	840 (72.8%)
Postgraduate	314 (27.2%)
Occupation	
Employed	587 (51.2%)
Currently unemployed	111 (9.6%)
Student	456 (39.5%)
Currently Smoking	
No	986 (85.4%)
Yes	168 (14.6%)
Chronic Disease	
No	900 (78.0%)
Yes	254 (22.0%)
Total SB (hours/day)	8.4 (5.5)
Mentally Active SB (hours/day)	5.6 (4.6)
Men	6.3 (4.6)
Women	5.2 (4.5)
Mentally Passive SB (hours/day)	2.5 (3.3)
Men	2.3 (3.1)
Women	2.6 (3.3)

BMI; body mass index, kg/m^2^; kilogram per meter squared, SB; sedentary behavior.

**Table 2:** Odds ratios of being overweight/obese per hour/day of SB in adults (n = 1154).

Variables	Total SB (h/day) *	Total SB (h/weekday) **	Total SB (h/weekend) **	Mentally Passive SB (h/day) ^#^	Mentally Passive S (h/weekday) ^##^	Mentally Passive SB (h/weekend) ^##^	Mentally Active SB (h/day) ^#^	Mentally Active SB (h/weekday) ^##^	Mentally Active SB (h/weekend) ^##^
Overall Sample									
OR	1.030	0.997	1.036	1.015	0.991	1.027	1.037	0.999	1.041
(95% confidence interval)	(0.999, 1.061)	(0.956, 1.038)	(0.995, 1.079)	(0.956, 1.077)	(0.917, 1.072)	(0.950, 1.110)	(0.998, 1.077)	(0.950, 1.051)	(0.988, 1.096)
Women									
OR	1.016	1.006	1.010	1.035	1.007	1.031	1.007	1.006	1.001
(95% confidence interval)	(0.976, 1.057)	(0.953, 1.063)	(0.957, 1.067)	(0.956, 1.061)	(0.905, 1.119)	(0.927, 1.146)	(0.956, 1.061)	(0.938, 1.079)	(0.933, 1.073)
Men									
OR	**1.052**	0.985	**1.076**	0.999	0.987	1.015	**1.075**	0.986	**1.107**
(95% confidence interval)	**(1.004, 1.102)**	(0.925, 1.049)	**(1.010, 1.146)**	(0.911, 1.096)	(0.876, 1.112)	(0.902, 1.142)	**(1.015, 1.140)**	(0.915, 1.036)	**(1.019, 1.202)**

CI, confident intervals, OR; odds ratio, SB; sedentary behavior. Bold indicates significant associations (*p* < 0.05), emphasizing variables with meaningful associations based on the binary logistic regression models; * indicates models adjusted for age, sex (for the whole sample), occupation, and chronic disease status; ** indicates models adjusted for age, sex (for the whole sample), occupation, chronic disease status, and, simultaneously, total SB during weekdays and weekends; ^#^ indicates models adjusted for age, sex (for the whole sample), occupation, chronic disease status, and, simultaneously, mentally active and passive SB per day; ^##^ indicates models adjusted for age, sex (for the whole sample), occupation, and chronic disease status and, simultaneously, mentally active and passive SB during weekdays and weekends.

**Table 3:** Isotemporal associations of replacing one hour of mentally active and passive SB with BMI in adults (n = 1154).

Variables	BMI (kg/m^2^)		
	β	SE	*p*-Value
Overall Sample			
Replacing mentally active SB with mentally passive SB (h/day)	−0.087	0.111	0.436
Replacing mentally active SB with mentally passive SB during (h/weekday)	−0.138	0.142	0.329
Replacing mentally active SB with mentally passive SB on (h/weekend)	0.075	0.145	0.603
Women			
Replacing mentally active SB with mentally passive SB (h/day)	0.080	0.135	0.552
Replacing mentally active SB with mentally passive SB during (h/weekday)	−0.172	0.179	0.337
Replacing mentally active SB with mentally passive SB on (h/weekend)	0.287	0.178	0.106
Men			
Replacing mentally active SB with mentally passive SB (h/day)	−0.245	0.183	0.181
Replacing mentally active SB with mentally passive SB during (h/weekday)	−0.019	0.224	0.932
Replacing mentally active SB with mentally passive SB on (h/weekend)	−0.245	0.236	0.299

β, beta coefficient; BMI, body mass index; kg, kilogram; m^2^, meter squared; SE; standard error. All models adjusted for age, sex (for the whole sample), occupation, and chronic disease status.

## Data Availability

Data are available upon request to the corresponding author.
